# Incidence and Implications of Q Waves After Acute ST-Elevation Myocardial Infarction in the Percutaneous Coronary Intervention Era

**DOI:** 10.31083/RCM49662

**Published:** 2026-10-10

**Authors:** Lihi Levi-Gofman, Shaul Atar, Gassan Moady

**Affiliations:** ^1^Azrieli Faculty of Medicine, Bar Ilan University, 1311502 Safed, Israel; ^2^Department of Cardiology, Galilee Medical Center, 2210001 Nahariya, Israel

**Keywords:** acute myocardial infarction, Q wave, percutaneous coronary intervention, outcome, readmission

## Abstract

**Background:**

Immediate coronary reperfusion is the cornerstone of therapy for acute ST-elevation myocardial infarction (STEMI), aiming to limit myocardial damage. Traditionally, the development of pathological Q waves on the electrocardiogram (ECG) has been considered a marker of irreversible transmural myocardial injury. However, most data on the incidence of pathological Q waves originate from the thrombolytic era. Therefore, the incidence and clinical significance of pathological Q waves in patients undergoing percutaneous coronary intervention (PCI) within the currently recommended time window remain unclear, as does the overall relationship with clinical outcomes.

**Methods:**

This study included 116 patients with STEMI who underwent PCI. In accordance with the current European Society of Cardiology guidelines, treatment was initiated within the recommended 90 minutes after admission.

**Results:**

Pathological Q waves developed in approximately 14% of patients, a lower incidence than that reported in the thrombolytic era. Pathological Q waves were more frequent among older patients, those with a lower baseline left ventricular ejection fraction (LVEF), and those with a longer time interval between admission and PCI. After adjustment for multiple variables, the presence of new pathological Q waves was not associated with an increased rate of one-year readmissions.

**Conclusions:**

In patients with STEMI undergoing PCI within the recommended time window, the incidence of pathological Q waves is substantially lower than that reported in the thrombolytic era and does not appear to be associated with a higher risk for readmissions. Further larger studies are warranted to confirm these findings.

## 1. Introduction

Acute myocardial infarction (AMI) remains a leading cause of morbidity and mortality worldwide despite major advances in percutaneous coronary intervention (PCI) [1]. AMI is classified based on the electrocardiogram (ECG) as ST-segment elevation myocardial infarction (STEMI) or non-ST-segment elevation myocardial infarction (NSTEMI), a distinction with important therapeutic implications, as STEMI is typically associated with complete coronary artery occlusion and requires urgent reperfusion. A definitive diagnosis of AMI requires laboratory confirmation of myocardial necrosis, typically indicated by elevated troponin levels, which provide both diagnostic and prognostic information [2]. Historically, pathological Q waves on an ECG have been considered markers of transmural myocardial necrosis and subsequent scar formation in the affected region [3,4,5,6]. Cardiac magnetic resonance imaging (CMR) studies have similarly shown that admission Q waves in patients with STEMI emerged as independent risk marker for microvascular injury [7]. Most studies evaluating the association between echocardiographic findings and Q-wave status on ECG were performed in the thrombolysis era [3,4]. The present study aimed to determine the incidence of new pathological Q waves in patients with STEMI treated with timely PCI within the recommended 90-minute window, and to evaluate whether Q-wave status was associated with clinical outcomes, including mortality and rehospitalization for cardiovascular events.

## 2. Methods

### 2.1 Study Population

This retrospective study included patients hospitalized with STEMI who were eligible for primary PCI. All patients aged >18 years who were admitted within 48 hours of symptom onset and were candidates for primary PCI were included. Patients were recruited from the database of individuals hospitalized with STEMI between January 2021 and July 2022. Of 137 patients, 5 were excluded for being ineligible for primary PCI (late admission), and 16 were excluded due to abnormal baseline Q waves on the ECG. Data were collected retrospectively from electronic medical records. Extracted variables included demographic characteristics (age and comorbidities); infarct location (anterior, inferior, lateral, or posterior wall); ECG findings (presence of new pathological Q waves); and echocardiographic parameters (specifically, left ventricular ejection fraction (LVEF)).

### 2.2 Echocardiography

All patients underwent echocardiography (Philips EPIQ 7 system with S5-1 transducer; Philips, Andover, MA, USA) within 1–2 days of hospitalization. LVEF was calculated using Simpson’s biplane method of disks based on apical two- and four-chamber views, using the formula: LVEF = (LVEDV − LVESV) / LVEDV × 100, where LVEDV and LVESV represent the left ventricular end-diastolic and end-systolic volumes, respectively.

### 2.3 Electrocardiogram

Electrocardiographic changes were documented for all patients at admission and after PCI. Pathological Q waves were defined as Q waves ≥40 ms in duration, ≥2 mm in depth, or ≥25% of the ensuing R-wave amplitude in at least two contiguous leads, or any Q wave or QS complex in leads V1–V3 [6,7].

### 2.4 Clinical Outcomes

For all patients, follow-up data were collected on all-cause mortality within 1 year and on hospital readmissions for recurrent cardiovascular events, defined as recurrent AMI (STEMI or NSTEMI) or unstable angina requiring PCI.

### 2.5 Statistical Analysis

Patient demographic data are presented as percentages and frequencies. Continuous variables are expressed as the mean ± standard deviation (SD) or as the median with interquartile range (IQR), as appropriate. Electrocardiographic data are reported as the presence or absence of Q waves according to established criteria for significant Q waves. Comparisons between patients with and without Q waves were performed using independent-samples *t*-test - for continuous variables and the chi-square test for categorical variables. Multivariable logistic regression was used to evaluate the correlation between Q waves and readmissions. A Kaplan–Meier curve was generated to depict 1-year readmission in the two patient groups. All statistical analyses were conducted using IBM SPSS Statistics software, version 27 (IBM Corp., Armonk, NY, USA).

## 3. Results

A total of 116 patients with STEMI (mean age 62 years, 86% male) were included in the final analysis. Baseline characteristics of the study population are presented in Table 1 .

**Table 1.** **Baseline characteristics of the study population**.

n		116
Age, mean ± SD		62 ± 10
Male, n (%)		100 (86)
Tobacco use, n (%)		73 (63)
Hypertension, n (%)		101 (87)
Diabetes mellitus, n (%)		89 (77)
CKD, n (%)		42 (36)
Hyperlipidemia, n (%)		107 (92)
Previous CAD, n (%)		81 (70)
Medications		
	Aspirin	89 (77)
	Statin	112 (97)
	Beta blockers	72 (62)
	ACE inhibitors	80 (69)

ACE, angiotensin-converting enzyme; CAD, coronary artery disease; CKD, chronic kidney disease; SD, standard deviation.

All patients underwent primary PCI within the recommended time window by current European Society of Cardiology (ESC) guidelines for the management of STEMI. Overall, thrombolysis in myocardial infarction (TIMI) 3 flow in the culprit artery was achieved in all patients at the end of PCI. Table 2 summarizes the STEMI subtypes and the main outcomes.

**Table 2.** **Infarct type and main outcomes**.

n		116
STEMI		
	Anterior, n (%)	59 (51)
	Inferior, n (%)	46 (40)
	Posterior, n (%)	7 (6)
	Lateral, n (%)	4 (3)
Time to PCI*, minutes (mean ± SD)		57 ± 18
Time to admission**, minutes (median, IQR)		45 (30–110)
Number of vessels, median (IQR)		2 (2–3)
LVEF, % (mean ± SD)		49 ± 10
Length of stay, days (mean ± SD)		3 ± 1
All cause mortality		4 (3)

IQR, interquartile range; LVEF, left ventricular ejection fraction; PCI, percutaneous coronary intervention; STEMI, ST-elevation myocardial infarction. * Time from admission to PCI. ** Time from onset of chest pain to admission.

New pathological Q waves, absent before primary PCI, were observed in 16 patients (14%). Compared with patients without Q waves, those with Q waves were older and had a lower baseline LVEF. Time from admission to PCI was significantly longer in patients who developed Q waves. These findings are summarized in Table 3.

**Table 3.** ** Baseline characteristics of patients with vs. without new Q waves**.

	Patients with Q waves	Patients without Q waves	*p*-value
	n = 16	n = 100	
Age, mean ± SD	69 ± 9	61 ± 10	0.0047
Male, n (%)	14 (88)	86 (86)	0.87
Hypertension, n (%)	13 (81)	88 (88)	0.43
Diabetes mellitus, n (%)	8 (50)	81 (81)	0.01
LVEF, % (mean ± SD)	41 ± 9	52 ± 10	0.0002
Anterior STEMI	5 (31)	54 (54)	0.07
Time to PCI	72 ± 22	55 ± 17	0.0005
TIMI flow 3	16 (100)	100 (100)	N/A

Readmission due to recurrent cardiovascular events (unstable angina or AMI) increased over time: 22% during the first year, 40% within three years, and 70% within five years. There was no statistically significant difference in the 1-year readmission rate between patients with and without new pathological Q waves (*p* = 0.8). Since overall mortality was low (3% of the entire cohort), no statistical analysis of mortality by Q-wave status was performed between the two groups. In multivariable regression, after adjusting for age, sex, hypertension, baseline LVEF, diabetes mellitus, time to PCI, and history of ischemic heart disease, the presence of new pathological Q waves was not independently associated with 1-year readmissions. Full model results are presented in Table 4.

**Table 4.** Multivariate regression for predicting readmissions^*^.

	Odds ratio (95% CI)	*p*-value
Age	1.01 (0.98–1.09)	0.13
Male	1.7 (0.27–9.3)	0.61
Hypertension	3.03 (0.53–17.4)	0.21
IHD	0.45 (0.14–1.18)	0.10
DM	2.0 (0.59–6.7)	0.27
Baseline LVEF	0.97 (0.92–1.02)	0.25
Time	1.01 (0.98–1.03)	0.62
Presence of Q wave	1.3 (0.33–5.02)	0.71

CI, confidence interval; DM, diabetes mellitus; IHD, ischemic heart disease; LVEF, left ventricular ejection fraction. Time: time from admission to revascularization. ^* ^Multivariate regression was performed using the Wald test.

One-year readmissions stratified by Q-wave status are shown in Fig. 1, which demonstrates no significant separation between the Kaplan–Meier curves for patients with and without new pathological Q waves.

**Fig. 1.**
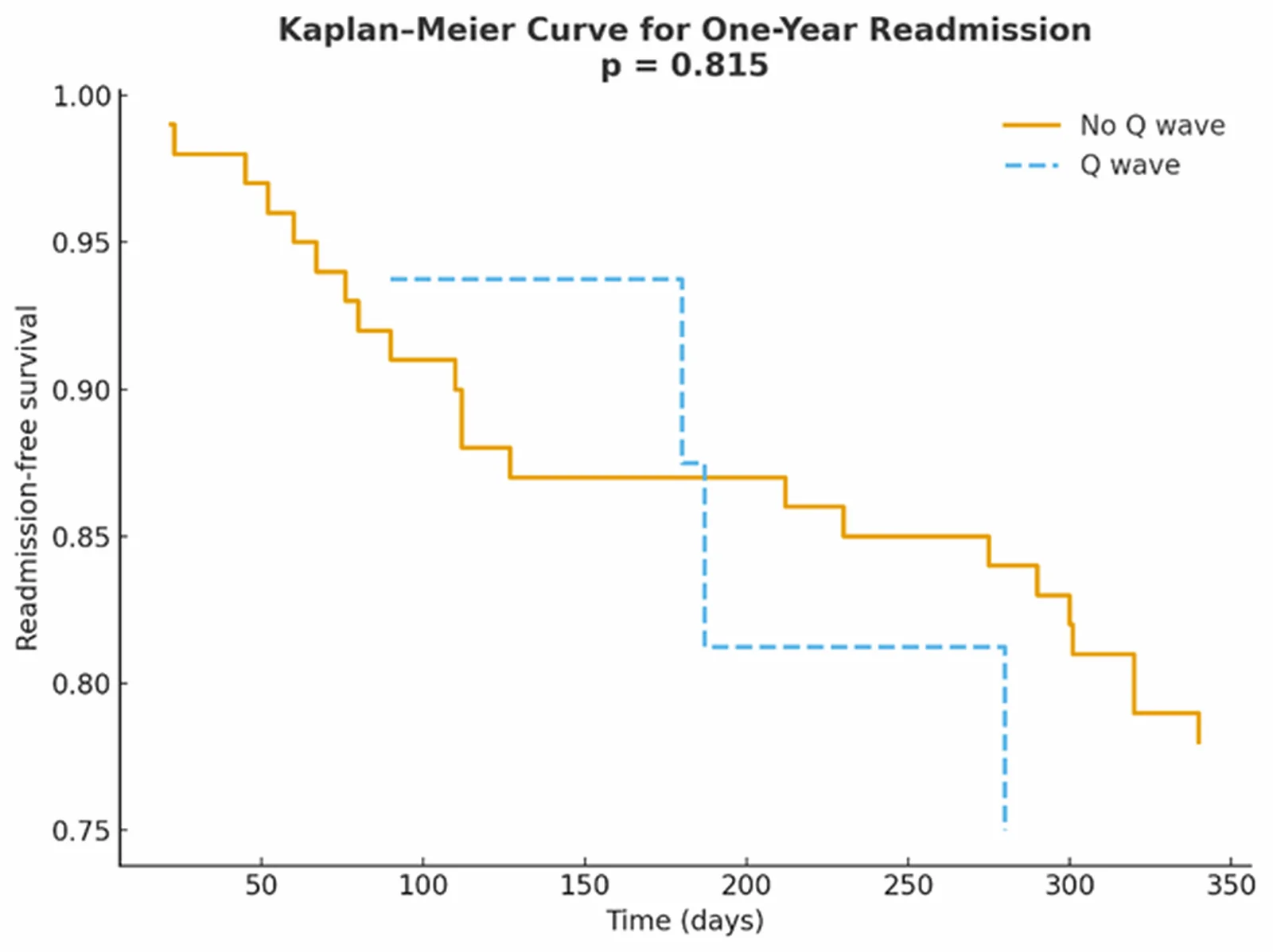
**Kaplan–Meier curve for 1-year readmissions stratified by Q**-**wave status**.

## 4. Discussion

This study evaluated the incidence of pathological Q waves after STEMI and examined their association with clinical outcomes in the contemporary PCI era. We found that 14% of patients developed new pathological Q waves, despite a mean time to PCI of 57 minutes, well within the ESC-recommended time window [8]. Older age, lower LVEF, and longer time from admission to PCI were associated with the development of pathological Q waves. However, the presence of new Q waves was not associated with an increased risk of 1-year readmission for cardiovascular events.

Previous registries have shown that the presence of baseline Q waves on admission in patients with STEMI is common, particularly among older individuals, and is associated with adverse outcomes [5,9]. Early Q waves have traditionally been associated with larger infarcts, lower LVEF, and a higher risk of mortality and heart failure compared with non-Q-wave AMI [9]. However, in the PCI era, the prognostic significance of new Q waves and their relationship with clinical outcomes remains uncertain. In addition to time to revascularization, advanced age, male sex, and incomplete reperfusion have been associated with a higher likelihood of Q-wave development [10,11]. The lower incidence of Q waves observed in the PCI era compared with the thrombolysis era likely reflects the higher rates of complete reperfusion achieved with catheter-based interventions, leading to smaller infarct sizes. Notably, in our study, LVEF assessed by echocardiography within the first two days after admission was largely preserved, and most patients underwent timely PCI, consistent with rapid revascularization.

Nevertheless, pathological Q waves still developed in a subset of patients. Traditionally, pathological Q waves on ECG have been regarded as irreversible markers of significant structural damage; however, in the modern era of early PCI, the prognostic value of Q waves and their reliability as a surrogate for precise imaging of myocardial function are increasingly questionable. CMR provides a more accurate assessment of myocardial damage and can detect even small areas of myocardial injury [7,12,13,14]. A lower incidence of Q waves was observed among diabetic patients in our cohort. This finding is unexpectable since diabetic patients often have atypical presentation of AMI, including symptoms such as dyspnea or generalized weakness, which can delay diagnosis and prolong the time to PCI, thereby increasing the likelihood of Q-wave development [15]. However, given the modest sample size, this observation should be interpreted cautiously and confirmed in larger cohorts.

Pathological Q waves following AMI arise from electrical changes within the infarcted myocardium. As necrotic tissue evolves into a fibrotic scar, the tissue loses its ability to conduct electrical impulses and becomes electrically silent, producing negative deflections on the ECG. During ventricular depolarization, the net electrical vector is directed away from the infarcted region, which further contributes to the development of Q waves in the corresponding leads. These changes are attenuated in promptly revascularized myocardium; thus, these are less common in the PCI era. Furthermore, new Q waves after PCI may reflect ischemic injury occurring before or during the procedure (*e*.*g*., periprocedural AMI) and may be transient in the setting of smaller infarcts or with rapid reperfusion, potentially resolving over time [16]. Thus, serial ECG follow-up is rational since early Q waves on presentation in patients with STEMI are not necessarily associated with poor outcomes [17]. Therefore, when assessing the incidence of new Q waves in post-AMI patients, it is important to consider the characteristics of the study population, total ischemic time, the specific criteria used to define pathological Q waves and their evolution over time, and alternative causes of Q-wave formation.

In summary, compared with historical thrombolysis-era cohorts, the incidence of pathological Q waves after STEMI in the PCI era appears lower when strict classic criteria are applied. However, contemporary estimates remain highly dependent on ECG timing and Q-wave definition. Thus, future studies should include serial ECG recordings at discharge and during follow-up (*e*.*g*., 30 days and 6–12 months), as well as comprehensive outcome measures including major adverse cardiovascular events.

## 5. Limitations

This study has several limitations. First, this study was a small retrospective analysis from a single center with a relatively limited sample size; thus, larger studies are needed to draw more definitive conclusions regarding the incidence of new Q waves after STEMI in the era of primary PCI. With 116 subjects, the study would have limited power to detect smaller effect sizes; approximately 690 patients would be required to achieve 80% power at a two-sided α of 0.05. Second, we assessed the time from hospital arrival to PCI, a widely used quality metric for STEMI care; however, this study did not analyze the time from symptom onset to PCI, which more accurately reflects the true duration of ischemia and may be more relevant in this context. However, precise assessment of symptom onset is prone to recall bias, particularly in the acute setting, and may introduce misclassification in both directions. Third, follow‑up ECGs beyond the index hospitalization were not routinely available. Consequently, we could not evaluate the temporal evolution or potential regression of Q waves over weeks to months, which may limit our ability to characterize their prognostic implications. Fourth, although TIMI 3 was achieved in all patients at the end of PCI, we did not collect data on myocardial blush grade, which is more accurate for evaluating myocardial reperfusion, and could represent an unmeasured confounder influencing Q-wave development.

## 6. Conclusions

The incidence of pathological Q waves in the era of primary PCI for STEMI is lower than that reported during the thrombolytic era. Older patients and those with lower LVEF appear to be at higher risk of developing Q waves. In the present study, new pathological Q waves were not associated with an increased risk of readmissions at 1 year.

## Data Availability

The datasets generated and analyzed during the current study are available from the corresponding author on reasonable request.
